# Engineering Stem Cell Recruitment and Osteoinduction via Bioadhesive Molecular Mimics to Improve Osteoporotic Bone-Implant Integration

**DOI:** 10.34133/2022/9823784

**Published:** 2022-09-06

**Authors:** Jiaxiang Bai, Gaoran Ge, Qing Wang, Wenming Li, Kai Zheng, Yaozeng Xu, Huilin Yang, Guoqing Pan, Dechun Geng

**Affiliations:** ^1^Department of Orthopaedics, The First Affiliated Hospital of Soochow University, 188 Shizi Street, Suzhou, Jiangsu 215006, China; ^2^Institute for Advanced Materials, School of Materials Science and Engineering, Jiangsu University, 301 Xuefu Road, Zhenjiang, Jiangsu 212013, China

## Abstract

For patients with osteoporosis, the therapeutic outcomes of osteoimplants are substantially affected by the impaired proliferation, migration, and osteogenic differentiation abilities of bone marrow mesenchymal stem cells (BMSCs). To improve bone-implant integration in osteoporotic condition, here we reported a one-step biomimetic surface strategy to introduce BMSC recruiting and osteoinductive abilities onto metallic osteoimplants. In our design, the bioadhesive molecular peptide mimic inspired by mussel foot proteins (Mfps) was used as molecular bridging for surface functionalization. Specifically, a BMSC-targeting peptide sequence (E7) and an osteogenic growth peptide (Y5) were grafted onto the titanium implant surfaces through a mussel adhesion mechanism. We found that a rational E7/Y5 feeding ratio could lead to an optimal dual functionalization capable of not only significantly improving the biocompatibility of the implant but also enabling it to recruit endogenous BMSCs for colonization, proliferation, and osteogenic differentiation. Mechanistically, the E7-assisted in situ recruitment of endogenous BMSCs as well as the enhanced interfacial osteogenesis and osteointegration was associated with activation of the C-X-C chemokine receptor type 4 (CXCR4) receptor on the cell surface and promotion of stromal cell-derived factor (SDF-1*α*) autocrine secretion. We anticipated that rational dual-functional surfaces through bioadhesive molecular mimics will provide a simple, effective, nonimmunogenic, and safe means to improve the clinical outcomes of intraosseous implants, especially under osteoporotic conditions.

## 1. Introduction

With the dramatic increase in age-related osteoporosis, bone loss associated with low bone turnover and decreased bone mass, the therapeutic results of medical osteoimplant surgery have been critically challenged [1, 2]. Titanium (Ti) and its alloys are one of the most popular orthopedic implant materials [3] owing to their excellent properties, including robust mechanical intensity, high corrosion, and wear resistance and biocompatibility [4]. However, the surface of the Ti-based material is not conducive for cells to adhere and grow due to a lack of bioactivity, especially for osteoblastic cells [5]. In addition, compared to normal individuals, the bone marrow microenvironment in osteoporosis is altered [6, 7], and the number and function of bone marrow mesenchymal stem cells (BMSCs) are decreased, which impairs bone formation, ultimately delaying bone repair and regeneration [8]. Therefore, it is difficult to achieve a stable bone-implant interface connection (i.e., osseointegration) for osteoporotic patients who eventually have prostheses implanted, which easily results in loosening and implant failure. Obviously, facilitating better bone-implant interface osseointegration requires much more than providing a cell-adhesive interface.

Recently, the utilization of in situ regeneration of bone tissue has been proposed [9], which may provide a rational strategy to promote osseointegration at the bone and implant interface under osteoporotic conditions. In situ bone tissue regeneration after implantation requires biomaterials to effectively recruit endogenous BMSCs and promote proliferation and differentiation, thus achieving interfacial bone tissue regeneration in situ [10]. Moreover, this approach involved no exogenous BMSCs and no ex vivo cell culture to avoid affecting signaling responses [11] and the homing capacity of BMSCs and decreased the risk of an intensive immune response and even infection [12]. Generally, the process of endogenous stem cell enrichment can be divided into two phases: the first is homing, defined as cell circulation, which drives BMSC migration from the bone marrow and peripheral blood vessels to the injury site; then, BMSCs are anchored at the surface of biomaterials to prevent them from flowing away by blood or fluids [13]. These two phases are critical to achieve enrichment of host stem cells at the surface of bone implants. After that, BMSCs proliferate and participate in the process of osteoblastic differentiation [14]. Consequently, an ideal implant surface should be decorated with desirable bioactivity that facilitates endogenous BMSC recruitment, adhesion, proliferation, and osteoinduction in situ and accelerates interface osseointegration, especially under aged osteoporotic conditions.

For Ti-based biomaterials, currently, multiple implant surface modification strategies have been utilized to facilitate specific cell responses (adhesion, proliferation, and differentiation) on the surface. Due to the excellent biocompatibility and its compositional similarity to bone mineral, hydroxyapatite (HA) surface modification has been widely used as an efficient strategy in load-bearing implants to enhance the osseointegration. [15]. However, due to the mechanical properties and its flexural strength and fracture toughness, HA coating can occur a fracture, exfoliation, and degradation, which limited the useful life of HA [16]. In addition, several other physical or chemical approaches were applied, including surface topological structures, growth factors [17], and proteins [18]. Nevertheless, these methods are also limited by their deficiencies. Briefly, the acid etching, alkali heat treatment technology used to construct the topological structure, is complicated and may change the inherent characteristics of the implant. Furthermore, traditional chemical fixation procedures are very complicated, and physical adsorption is nonspecific and inefficient. Most importantly, they are not suitable for all surfaces on various medical bone implants. With this in mind, we turned our attention to biomimetic strategies. Enlightened by the anchoring property of marine mussels on various surfaces, DOPA (3,4-dihydroxy-L-phenylalanine), which is abundant in Mytilus edulis foot proteins (Mefps), has attracted extensive attention [19–21]. A previous study showed that DOPA exhibits strong adhesion on the surface of wet metals through interactions between covalent cross-linking units, metal bidentate coordination, and hydrogen bonding regulated by catechol groups [22]. A specific example is the powerful coordination between the catechol ligand and negatively charged oxide units [23]. Moreover, a new titanium oxide layer could easily cover the surface of Ti and its alloys by immersion in mimicked physiological fluids [24]. Complementarily, DOPA is considered to have good durability for long-term operation in aqueous environments [25]. For example, Xi et al. reported that the DOPA coating was soaked in deionized water at 60°C for 36 days and washed for a long time, and its hydrophilicity was still well maintained [26]. Therefore, compared with conventional chemical and physical conjugations, rational modification of Ti implants with catechol groups might provide a more promising strategy to enhance adhesion.

**Figure 1:**
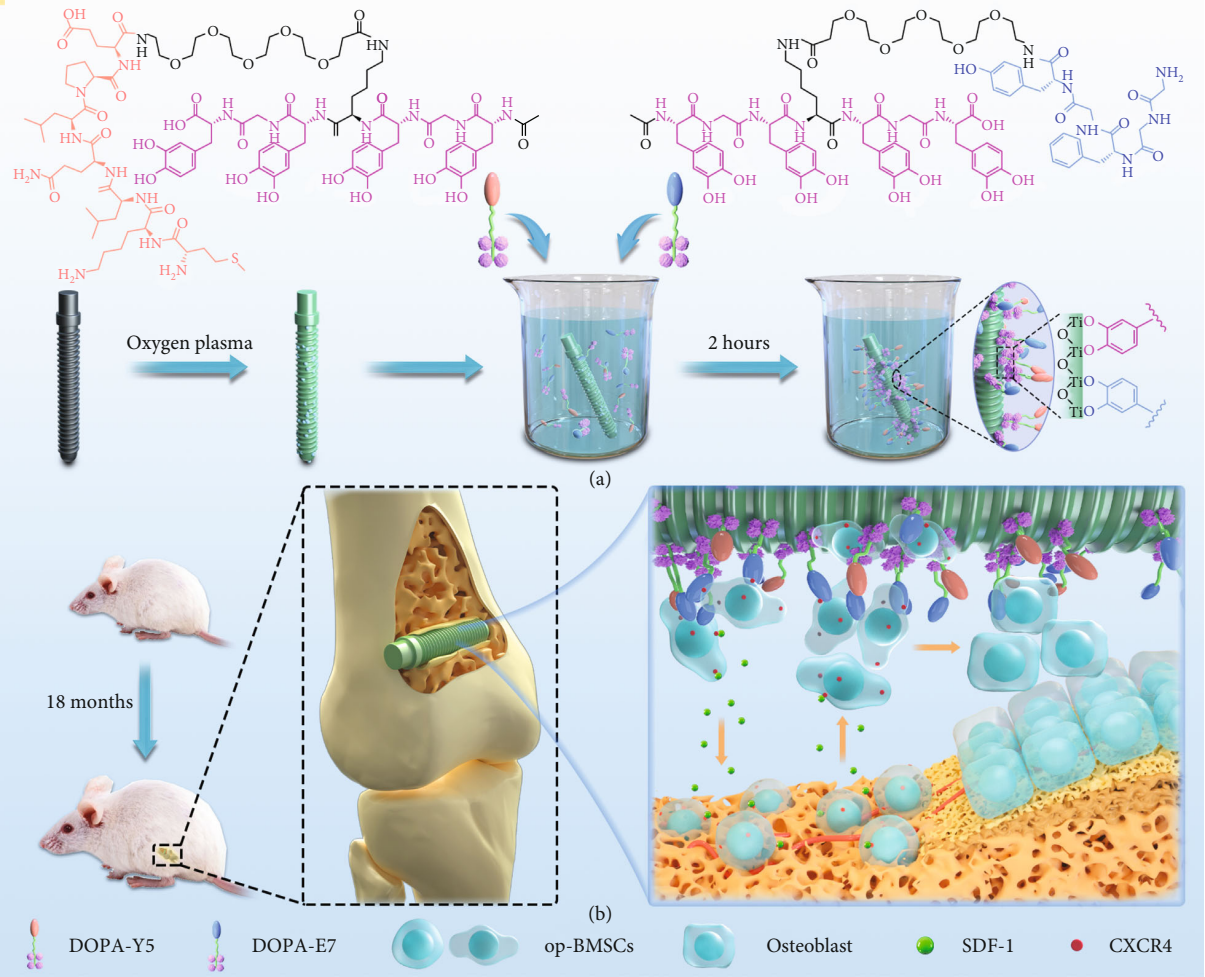
(a) Conceptual graphic of the mussel-inspired peptide on a medical Ti osteoscrew. (b) In a bone implantation model of aged osteoporotic rats, the DOPA-E7 and DOPA-Y5 peptide-modified Ti screws showed endogenous stem cell recruitment and osteoinductive dual functions in vivo, synergistically enhancing interfacial in situ osteogenesis and intrabone implant integration after implantation.

Based on the above problems, in this study, two bioactive peptides inspired by marine mussels, (DOPA)_4_-PEG_5_-EPLQLKM and (DOPA)_4_-PEG_5_-YGFGG, were employed to modify the bioactivities of Ti implants. These two biomimetic peptides both contained four catechol-DOPA sequences, five polyethylene glycol sequences, and a bioactive peptide segment. Of these, EPLQLKM (E7) is a heptapeptide that promotes the adhesion and proliferation of recruited stem cells based on its specific affinity [27]. YGFGG (Y5) is a bioactive peptide that originates from osteogenic growth peptides and regulates osteogenic differentiation [28]. This response indicated that, theoretically, the modification of Ti implants with the combined use of E7 and Y5 peptides could enhance endogenous BMSC enrichment and osteogenic differentiation for polyfunctionalization. Herein, we demonstrated for the first time that endogenous BMSCs respond to modified Ti surfaces under aged osteoporotic conditions, especially the multifunctionalized impact of Ti implants on osseointegration. Our findings may provide a promising new strategy to improve the clinical results of implantation in osteoporotic patients by the combined use of bioactive mimetic peptides to induce bone regeneration in situ (Figure 1).

## 2. Results and Discussion

### 2.1. Mussel-Inspired Peptide Synthesis and Surface Modification

**Figure 2:**
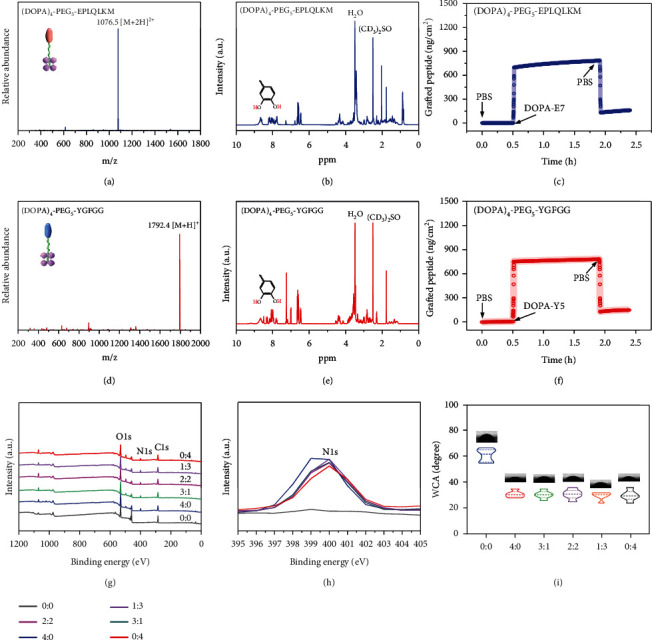
Characterization of the two biomimetic peptides and their adhesion to Ti-based implants. (a and d) ESI-MS spectra of DOPA-E7 and DOPA-Y5. (b and e) ^1^H NMR spectra of DOPA-E7 and DOPA-Y5. (c and f) DOPA-E7 and DOPA-Y5 cografting processes on TiO_2_ chips. (g) XPS analysis of different surfaces. (h) Changes in the N 1 s signal in the XPS spectrum. (i) The contact angle between water and different surfaces, as well as the quantitative results.

In accordance with our previously published method, we manually synthesized the mussel-inspired peptide via solid-phase peptide synthesis [29, 30]. Briefly, a commercially available acetonide-protected Fmoc-DOPA (acetone)-OH and a long polyethylene glycol (PEG) were utilized by introducing the catecholamine amino acid DOPA into the sequence of peptides in SPSS. In addition, acetyl groups were added to the N-terminus of the final peptides. Purified by high-performance liquid chromatography (HPLC, purity > 95%, Figure S1), electrospray ionization mass spectrometry (ESI-MS) was then utilized to analyze the characterizations of the two synthesized mussel-inspired peptides. The monoisotopic mass [M + H]^+^ of (DOPA)_4_-PEG_5_-EPLQLKM (noted as DOPA-E7) and (DOPA)_4_-PEG_5_-YGFGG (noted as DOPA-Y5) was measured at 2151.0 and 1791.4, which corresponded to their theoretical molecular weights of 2150.4 and 1791.8, respectively (Figures 2(a) and 2(d)). The chemical constructions of the two mussel-inspired peptides were detected by ^1^H NMR. An indication of the presence of DOPA units was clearly visible with a diagnostic peak at 8.65 ppm that referred to catecholic hydrogens (Figures 2(b) and 2(e)). These results suggested that we successfully synthesized the two biomimetic peptides with multiple DOPA units and different bioactive motifs.

After obtaining the two peptides, the DOPA-E7 and DOPA-Y5 peptide-commodified surface was prepared by surface coating on a Ti-based substrate. Studies have demonstrated previously that multivalent catechol-containing molecules can be easily and stably grafted onto Ti-based oxides through coordination [31]. Therefore, DOPA-E7 or DOPA-Y5 coatings were prepared by incubating the Ti plates in phosphate-buffered saline solution (PBS, 0.02 mM, pH = 7.2) with the two mussel-inspired bioactive peptides. In the initial experiments, peptide binding and biomolecular grafting were monitored using a quartz-crystal microbalance (QCM). As shown in Figures 2(c) and 2(f), DOPA-E7 or DOPA-Y5 showed steady binding onto the QCM chips and reached maximal binding capacities of 782.8 ng·cm^−2^ and 782.5 ng·cm^−2^, respectively. In addition, surface elemental compositions with different feeding molar ratios of DOPA-E7 and DOPA-Y5 (i.e., 4 : 0, 3 : 1, 2 : 2, 1 : 3, and 0 : 4) were then characterized by X-ray photoelectron spectroscopy (XPS) to further verify peptide immobilization. As shown in Figures 2(g) and 2(h), an obvious enhancement in the N 1 s signal at 400.12 eV (corresponding to the amide in peptide bonds) was found on the grafted surfaces with mussel-inspired bioactive peptide. These results, including the detection of water contact angles under static conditions with different modifications (Figure 2(i)), clearly demonstrated bioactive peptide immobilization on Ti substrate surfaces.

### 2.2. Isolation, Identification, and Characterization of BMSCs

Despite the fact that BMSCs have excellent regenerative capabilities in various tissues, the characteristics of BMSCs under pathological conditions differ significantly [32]. Previous in vitro studies of regenerative medicine mainly used normal-state BMSCs or MC3T3-E1 preosteoblast cell lines, ignoring the importance of directly studying BMSCs with impaired functions under pathological conditions [33, 34]. Therefore, we first confirmed the successful establishment of aged osteoporosis (18-month-old male rats) by 3D reconstruction of micro-CT (Figures S2(a)). In the osteoporotic rat group, the trabecular bone structure was less disordered, and the marrow cavities were enlarged. Compared with the normal control group (3-month-old male rats), bone mineral density (BMD), relative bone volume/tissue volume (BV/TV), and trabecular thickness (Tb. Th) in the osteoporosis group were significantly decreased (Figures S2(b)). Additionally, there was a greater than 1.3-fold increase in the trabecular space (Tb. Sp) as well. These results indicate that the age-related osteoporotic rat model was well developed.

Then, the BMSCs were isolated from the above developed osteoporotic rats and displayed a typical immunophenotype, denoted op-BMSCs (Figure S3). Briefly, flow cytometry assays (FACs) revealed that op-BMSCs were consistently free of hematopoietic cells and did not express CD34 or CD45 but expressed markers associated with BMSCs, including CD90 and CD29. To further characterize op-BMSCs, proliferation, migration, and osteoblastic differentiation assays were performed. The assay for counting cells using the cell counting kit-8 (CCK-8), scratch experiment, alkaline phosphatase (ALP), and alizarin red (ARS) staining results indicated that op-BMSCs exhibited significantly weaker proliferative, chemotaxis, and osteogenesis abilities (Figure S4). Therefore, it is extremely important to create a favorable environment for the proliferation, chemotaxis, and osteoblastic differentiation of op-BMSCs by modifying medical osteoimplants.

### 2.3. In Vitro Cytocompatibility Evaluation of Peptide Coating

Biocompatibility is a prerequisite for any medical implant biomaterial [35]. In this regard, a number of in vitro studies have been conducted using op-BMSCs to examine cell adhesion and proliferation on Ti-based substrates before and after surface functionalization. Live/dead staining showed that the two mussel-inspired peptides with different feeding molar ratios (DOPA − E7 : DOPA − Y5 = 4 : 0, 3 : 1, 2 : 2, 1 : 3, and 0 : 4) obviously reduced dead cells compared to the bare Ti group (Figure S5). Then, a CCK-8 assay was employed to evaluate the proliferation of op-BMSCs. The results showed that all the peptide-treated groups showed significant proliferation and viability for op-BMSCs compared with the bare Ti group (Figure S6(a)). In addition, at 2 and 4 hours after cell implantation, counts of adherent cells were performed. The number of cells attached to the surfaces of DOPA-E7-containing Ti-based surfaces was higher than that attached to the bare-Ti surfaces following incubation (time: 4 h) (Figure S6(b)). The results suggested that the DOPA-E7-containing surface increased cell anchorage efficiently. Notably, slightly higher adhesion was also observed on the DOPA-Y5-treated Ti-based surface than on the untreated surface, probably due to the multivalent interactions between DOPA-Y5 peptides and the corresponding receptors on the cell membrane.

To further identify the effect of the different surfaces on cell adhesion behavior, the cytoskeleton staining and SEM scanning in the cells cultured on different samples were assessed. Cytoskeleton staining showed that op-BMSCs on the bare Ti substrate exhibited a relatively spherical morphology after 24 hours of culture (Figure S7(a)). In contrast, the peptide-modified surfaces exhibited better adhesion with polygonal shapes and increased filamentous F-actin expression. Meanwhile, SEM images showed that op-BMSCs on the bare Ti substrate surface exhibited less spreading than cells on the peptide-coated surfaces (Figures S7(b)), which is largely consistent with the results of cytoskeleton staining. The above results demonstrated that the two bionic peptide-modified surfaces had a positive effect on the adhesion and proliferation of op-BMSCs, which would be an environment that promotes tissue regeneration.

### 2.4. In Vitro op-BMSC Recruitment

**Figure 3:**
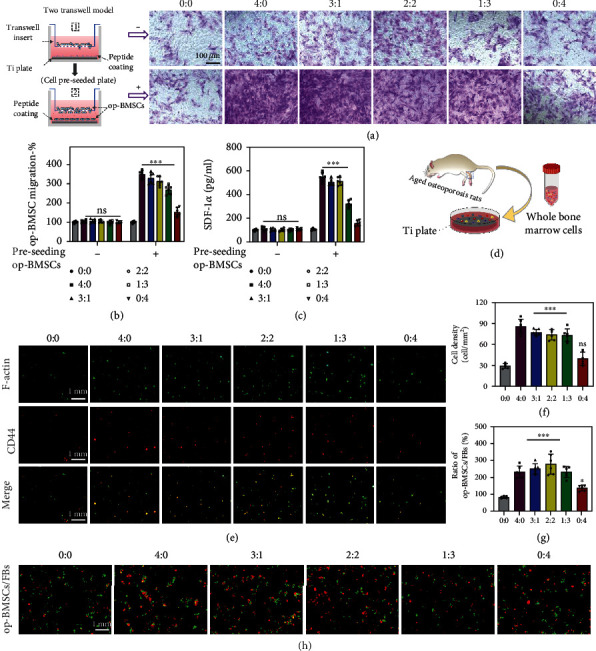
In vitro op-BMSC recruitment. (a) Before or after preseeding cells on sample surfaces, transwell assays highlighting op-BMSC recruitment in the upper chambers of various feeding ratios (DOPA − E7 : DOPA − Y5 = 0 : 0, 4 : 0, 3 : 1, 2 : 2, 1 : 3, and 0 : 4). (b) Quantitative analyses of migrating cells. (c). ELISA-determined SDF-1*α* level. (d) Schematic showing whole bone marrow cells cultured on the Ti surface after extraction from femurs in aged osteoporotic rats. (e and f) The six different Ti surfaces were cultured together with the whole bone marrow. Three days later, the recruitment of op-BMSCs to the Ti surfaces was confirmed by CD44 immunofluorescent staining (red: CD44, green: FITC-phalloidin). (g and h) Competitive growth and correspond quantitative of op-BMSCs (green) to fibroblasts (red) on different surfaces. (*n* = 6; ns: not significant; ^∗∗^*P* < 0.005 and ^∗∗∗^*P* < 0.001 compared with the bare surface).

Efficient in situ bone formation requires the recruitment, colonization, and osteogenic differentiation of BMSCs. Of these, cell recruitment begins first with stem cell migration [36, 37]. Therefore, we first evaluated the migration-inducing effects of the six different groups of sample surfaces on op-BMSCs via a transwell migration assay (Figure 3(a)). Contrary to expectations, we found that the op-BMSCs that migrated on the lower surface of all the membranes showed almost no significant difference. However, upon the addition of op-BMSCs on the sample surface, all the DOPA-E7-containing samples (i.e., 4 : 0, 3 : 1, 2 : 2, and 1 : 3) could induce more op-BMSCs to adhere to the membrane's lower surface than the other groups. Cell migration on the bare surface (0 : 0 group) showed no changes regardless of op-BMSCs supplementation. Quantitative analysis further confirmed this finding (Figure 3(b)). These results indicated that the mussel-inspired DOPA-E7 peptide coating could recruit significantly more op-BMSCs, and this may be due to the secretion of certain chemokines after contact with cells.

It has been reported that several chemokines contribute to recruiting MSCs to the surface/interface of implantation sites; examples include stromal cell-derived factor-1*α* (SDF-1*α*)/CXCR4 and CXCL13/CXCR13 [38, 39]. Among these, SDF-1*α* acts as a powerful chemoattractant for MSC migration from the stem cell niche to the injured site and participates in endogenous endochondral ossification. Therefore, we further detected the content levels of SDF-1*α* by ELISA. The results revealed no significant change among all groups when no additional cells were added, while SDF-1*α* secretion exhibited a remarkable upregulation in the DOPA-E7-containing samples in a concentration-dependent manner when additional op-BMSCs were added (Figure 3(c)). These results indicate that there is a good chance that SDF-1*α* was an essential component in the process of DOPA-E7-induced migration and differentiation of op-BMSCs. Therefore, this is a process that promotes the autocrine activity of endogenous MSCs, not the traditional way of loading exogenous cytokines.

Next, a 72-hour incubation period with a whole bone marrow cell suspension was conducted on these six groups of Ti surfaces (Figure 3(d)). Subsequently, nonadherent cells were removed from the Ti surfaces by thoroughly rinsing them. A phenotypic analysis of bound op-BMSCs was conducted by staining them for F-actin, which represents the cytoskeleton, and for CD44, which represents stem cells. As shown in Figures 3(e) and 3(f), each group induced recognizable adhesion of op-BMSCs (CD44+), and the groups with higher DOPA-E7 feeding (e.g., 4 : 0, 3 : 1, and 2 : 2) showed obviously stronger BMSC-capturing activity than the others. Previous studies have confirmed that fibrous tissue-like pseudomembranes are often formed at the loosened implant-bone interface, and fibroblasts (FBs), which account for 15%-30% of the pseudomembranous tissue, play an important role [40]. Specifically, a large number of fibroblasts accumulate to activate and secrete inflammatory factors to act on osteoblasts, weakening the osteogenic ability of osteoblasts. Therefore, we performed a coculture of op-BMSCs and fibroblasts to determine whether and how these two cell types exhibit competitive adhesion and growth. As shown in Figures 3(g) and 3(h), the ratios of op-BMSCs to fibroblasts adhered onto the peptide-treated Ti substrates all showed a significant increase compared with those of the controls, and the higher DOPA-E7 feeding ratio (i.e., 4 : 0, 3 : 1, and 2 : 2) led to higher op-BMSC recruitment.

### 2.5. In Vitro Osteogenesis

**Figure 4:**
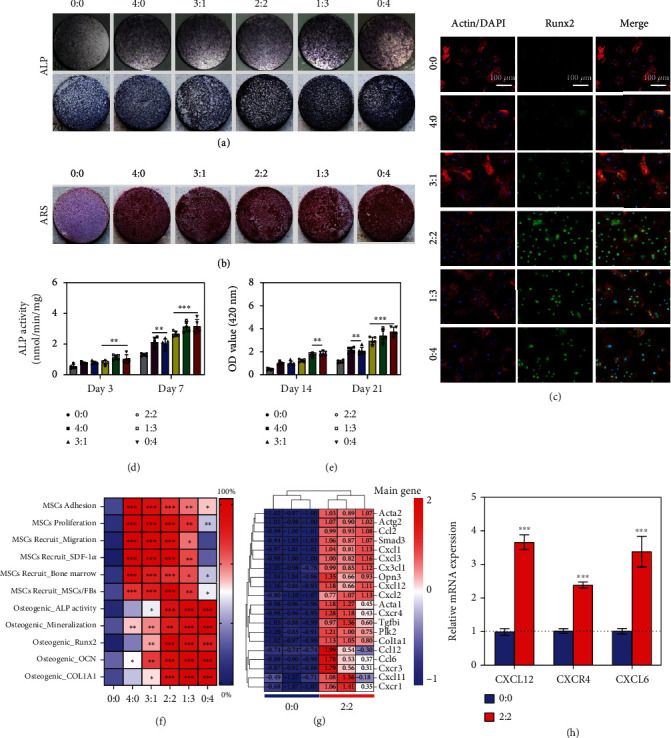
The mussel-inspired peptide-coated Ti surfaces exhibited enhanced osteogenesis. (a) Representative general images of ALP staining on op-BMSCs cultured on different surfaces after 3 and 7 days. (b) Representative images of ARS staining after 21 days. (c) Images of the op-BMSCs after immunofluorescent staining (green: Runx2; red: cytoskeleton; and blue: nuclei). Quantitative ALP activity (d) and mineral layer (e) of op-BMSCs on different surfaces on the indicated days. (f) Heat map of the standardized expected performance of peptide recruitment and osteogenic differentiation. (g) Heat map of genes associated with the cell migration pathway. (h) Real-time PCR of CXCL12, CXCR4, and CXCL6 mRNA expression in op-BMSCs. (*n* = 3; ^∗∗^*P* < 0.005 and ^∗∗∗^*P* < 0.001 compared with the bare surface).

Next, the effect of the abovementioned biomimetic peptide-modified titanium surface on cell osteogenesis was investigated. Considering that the osteogenic capacity of the aging in vivo environment is weakened and the op-BMSCs are more inclined for bone-forming induction in the defect area, in this study, differentiation was carried out in vitro using an osteoinduction medium. This will be an improved design to analyze the effects of our mussel-inspired bioactive peptides on bone formation *in vitro.* First, ALP staining and activity were performed. After 3 and 7 days of osteogenic induction culture, all Ti surfaces showed identifiable ALP coloration, but it was clearly observed that a high proportion of DOPA-Y5 had a significantly deeper blue color on day 7 than in the other groups (Figure 4(a)). This may be related to the enhancement of this osteogenic effect in the later stage of differentiation. Quantitative analysis of protein also showed that compared with the low-concentration DOPA-Y5 group (i.e., 0 : 0, 4 : 0, and 3 : 1) and the higher DOPA-Y5 feeding ratio group (i.e., 2 : 2, 1 : 3 and 0 : 4), the ALP activity was stronger (Figure 4(d)), which is consistent with the ALP staining, but no significant differences exist between the three groups. After 21 days of osteogenic induction, the cell matrix mineralization ability on different surfaces was evaluated by alizarin red staining. Except for the control group, all other groups showed significantly increased intracellular matrix mineralization (Figure 4(b)), and bone mineralization also showed a DOPA-Y5 ratio-dependent effect (Figure 4(e)). Furthermore, the expression of proteins, including Runx2 (Figure 4(c)), in cells was analyzed by immunofluorescence after 4 days of osteogenic induction. Three groups (i.e., 2 : 2, 1 : 3, and 0 : 4) had significantly higher fluorescence intensities than the others, and Runx2 expression in the 2 : 2 group was the highest. In addition, as shown in Figure S8, the expression of all 4 kinds of osteogenic differentiation-related genes (Col1A1, OCN, Runx2, and ALP) was increasingly regulated in the DOPA-Y5-containing groups in comparison with the other groups, which corroborated the DOPA-Y5-dependent osteogenic activity. Taken together, enhanced osteogenic ability was demonstrated in vitro in groups containing DOPA-Y5.

### 2.6. Comprehensive Analysis and Molecular Mechanism Detection In Vitro

Through an all-encompassing evaluation of the above in vitro results, it is clear that the mussel-inspired multifunctional surface modification strategy used for titanium implants is effective. On the one hand, increasing the ratio of DOPA-Y5 is beneficial to the osteogenic ability of op-BMSCs. However, increasing the proportion of DOPA-E7 on the titanium surface facilitated the recruitment of op-BMSCs. Therefore, to obtain the best titanium implant surface, we first standardized the expected performance of peptide recruitment and osteogenic differentiation and then summarized all the in vitro results as a heat map (Figure 4(f)). The coating group with a significant difference compared with the blank control group is marked with an asterisk. As shown in the heat map, the cografted surface with a DOPA-E7/DOPA-Y5 feeding ratio of 2 : 2 had the best overall effect. It effectively captures stem cells and promotes stem cell adhesion, proliferation, and osteogenic differentiation. Therefore, we chose this optimized ratio (DOPA − E7 : DOPA − Y5 = 2 : 2) for the functional modification of the surface of medical Ti screws to evaluate the interface in situ bone regeneration in aged osteoporotic rats in vivo (Figure S9).

To identify the potential molecular mechanisms underlying host op-BMSCs interactions with the peptide coating, the expression profiles of genes were compared by RNA sequencing (RNA-seq) of op-BMSCs cultured with the mussel-inspired peptide coating. According to gene expression analysis, 1564 genes were upregulated in op-BMSCs in the coating group, and a total of 1289 genes were downregulated (*P* *value* < 0.05 and |log2*FC*| > 1) compared with the bare surface (0 : 0 group) (Figure S10(a)). GO analysis results showed that the upregulated and downregulated genes were involved in chemokine-mediated signaling pathways, biological adhesion, bone mineralization, protein binding, cell junction formation, biological regulation, and processes (Figures S10(b) and (c)). Among them, genes associated with the cell migration pathway, including ACTA1, ACTG2, CXCR4, CXCL1, and CXCL12 (also known as SDF-1*α*), were prominently enhanced by peptide coating (Figure 4(g)). Pathway analysis of the upregulated genes by Kyoto Encyclopedia of Genes and Genomes (KEGG) revealed that interactions between cytokines and their receptors and chemokine signaling pathways might mediate BMSC migration (Figure S11). Moreover, we found that the rank of core genes CXCR4 and CXCL12 was highest by screening out hub genes of the top 25 KEGG pathways (Figure S12). The mRNA expression of CXCL12, CXCR4, and CXCR6 in op-BMSCs was also confirmed to be increased in the coating group, suggesting that CXCR4-CXCL12 plays a significant role in this process (Figure 4(h)). Together with the previously described findings, these results illustrate that the DOPA-E7 peptide coating might promote op-BMSC migration by modulating CXCR4-CXCL12 chemokine signaling pathways. That is, after DOPA-E7 binds to the CXCR4 receptor on the surface of BMSCs, it causes the cells to autocrine SDF-1*α*, and then, the secreted SDF-1*α* can bind to the CXCR4 receptor on the surface of other stem cells in the distance, thus forming a chemical cascade reaction, continuously recruiting endogenous stem cells. However, research on the exact mechanisms involved in chemokine pathway activation remains to be done.

### 2.7. In Vivo Cell Recruitment

**Figure 5:**
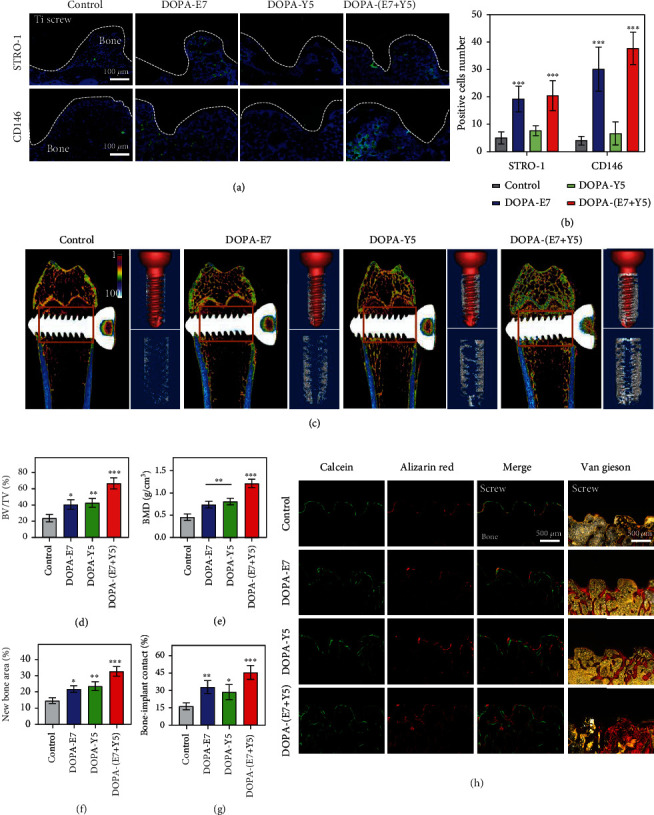
The mussel-inspired peptide-treated screws exhibited enhanced BMSC recruitment and osseointegration in vivo compared to the untreated screws. Immunofluorescence (a) and semiquantitative (b) analyses of STRO-1 and CD146 expressions in the bone-screw interface at 7 days after implantation. (c) Images of micro-CT 3D reconstruction and quantitative evaluation of peri-implant bone formation (parameters including (d) BV/TV and (e) BMD). (f and h) Calcein-ARS staining and Van Gieson staining for the newly formed bone and quantitative staining analysis. (*n* = 6; ^∗∗^*P* < 0.005 and ^∗∗∗^*P* < 0.001 compared with the bare surface).

Intrinsic BMSCs are the source of osteoblasts in bone [41]. Therefore, the impacts of the bionic coating on the recruitment of BMSCs in vivo were evaluated. Animal experiments were conducted using a drill with a diameter between concave and convex screws to minimize surface coating destruction during implantation. Titanium screws were first implanted in the distal femur of aged osteoporotic rats. One week later, the accumulation of op-BMSCs at the bone-screw interface was studied and identified by CD146 and STRO-1 immunofluorescence staining. As seen from Figures 5(a) and 5(b), there were only a small number of CD146-positive and STRO-1-positive cells in the interface tissues of the control group and the DOPA-Y5 group. Compared with this, the number of positive cells in the DOPA-E7 group and DOPA-(E7+Y5) group increased significantly. Overall, the grafting of E7 active peptides to the surface of bone implant materials by DOPA, followed by directional recruitment of BMSCs, is a prerequisite for bone interface integration. This process of recruiting BMSCs in situ is of great interest, as this process enables bone regeneration without replenishing exogenous cells.

### 2.8. Osseointegration In Vivo

Next, 8 weeks after implantation in vivo, changes in bone formation and osseointegration were evaluated at the interface between the screw and bone tissue. The collected bone tissue was subjected to micro-CT scanning/3D reconstruction and histological analysis.  Figure 5(c) shows that the DOPA-(E7+Y5) group produced the most newly formed bone tissue around the screw, while the bare surface (control) group only covered some sporadically broken bone tissue. An analysis of the quantitative data further confirmed this conclusion (Figures 5(d) and 5(e)). For example, the DOPA-(E7+Y5) group of screws had the highest BV/TV percentage and exhibited the best trabecular structure in the same region of interest. These results are due to the synergistic effect of the op-BMSC recruitment peptide and the osteogenic growth peptide, and a single modification (DOPA-E7 or DOPA-Y5)-provided microenvironment may not be optimal for interface bone regeneration under osteoporosis. Next, continuous fluorescent labeling of new bone with calcein and ARS dual fluorescent labeling and Van Gieson staining yielded consistent results (Figures 5(f) and 5(h)). By Van Gieson staining, we clearly observed large areas of new bone mineralization on the interface in the DOPA-(E7+Y5) group (32.5% ± 2.61%) and slightly increased bone mineralization in the DOPA-E7 (21.6% ± 1.85%) and DOPA-Y5 (23.4% ± 2.42%) groups compared with the control group (14.5% ± 1.55%). Quantitative analysis also showed that the BIC for the DOPA-(E7+Y5) group was significantly higher than that for the other groups.

**Figure 6:**
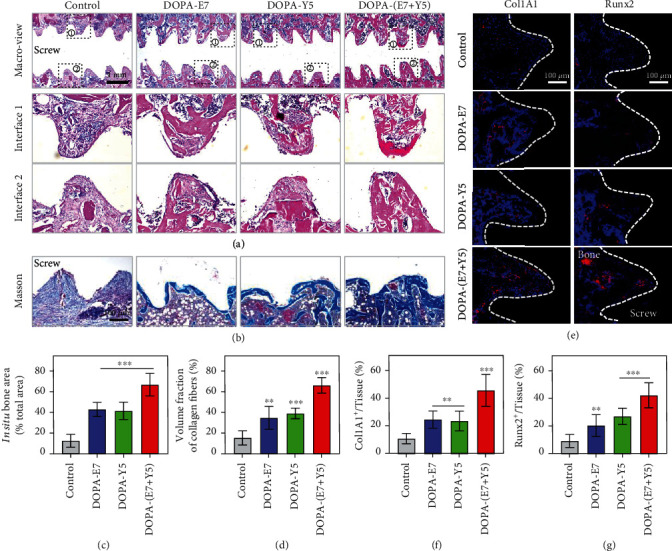
The mussel-inspired peptide-treated screws exhibited enhanced osteogenic differentiation in vivo. (a) H&E staining images of the peri-implant tissue and quantified with (c) in situ bone area; (b) Masson's trichrome staining exhibiting the new bone formation to the bone-implant interfaces and (d) quantified with volume fraction of collagen fibers. (e) Immunostaining images of the peri-implant tissue: red (Runx2 or Clo1A1) and blue (nuclei) and (f and g) quantitative positive cells as a proportion of total cells. (*n* = 3; ns: not significant; ^∗∗^*P* < 0.005 and ^∗∗∗^*P* < 0.001 compared with the bare surface).

To further analyze the regenerated bones, we performed H&E and Masson's trichrome staining. As shown in Figures 6(a) and 6(c), there was a large amount of fibrous connective tissue and little new bone formation in the interface area of the control group. However, in the DOPA-E7 and DOPA-Y5 groups, an increase in bone regeneration was observed around the area of implantation. Additionally, there were filled fully fused bone tissues in the bone-implant interface area of the DOPA-(E7+Y5) group. Masson's staining clearly showed that the interfaces were connected by a thin pale blue layer of fibrous connective tissues in the control group, and a small amount of new regenerated bone could be seen on the edge of the interface in the DOPA-E7 and DOPA-Y5 rats (Figures 6(b) and 6(d)). There was a greater degree of maturation and calcification of bones in the DOPA-(E7+Y5) rats. Additionally, the gene expression of the osteogenic transcription factor Runx2 and osteogenic marker Col1A1 in the DOPA-(E7+Y5) group was higher than that in the other groups (Figures 6(e) and 6(g)). The above results suggested that DOPA-(E7+Y5) peptide-coated Ti implants could promote interfacial bone regeneration and repair in aged osteoporotic rats.

## 3. Conclusion

In conclusion, we designed two novel mussel-derived bioactive peptides for the biological modification of Ti-based implants by a simple “one-step” method. Specifically, high biomimetic peptides with the BMSC affinity E7 sequence or Y5-derived osteogenic sequence as the upper limit can effectively improve the biocompatibility of Ti-based implants and endow the osteoimplants with desired biological activities, including the promotion of endogenous BMSC chemotaxis, adhesion, colonization, proliferation, and osteogenic differentiation. Mechanistically, the recruitment of endogenous BMSCs by the biomimetic coating was associated with activation of the CXCR4 receptor on the cell surface and promotion of SDF-1*α* autocrine secretion. In addition, it was demonstrated that the synergistic effect of osteogenicity and osseointegration of osteoimplants in vivo was significantly enhanced by using two biomimetic peptides in a rational ratio. Therefore, the highly biomimetic mussel-derived peptides and the dual-functional strategy in this study provide facile, rational, safe, and feasible surfaces for achieving effective in situ bone regeneration and improving the clinical outcome of medical implants, especially in the presence of an osteoporotic state.

## 4. Materials and Methods

### 4.1. Preparation and Synthesis of Materials

All cell culture plates were obtained from NEST Biotechnology. Ti-based plates and screws were custom ordered from Ideal Medical Appliance Co., Ltd. (Suzhou, China). The Ti-based materials were pretreated with oxygen plasma for 10 min to activate the bioactivity of the surface. After that, the Ti-based materials were immersed in (DOPA)4-PEG5-EPLQLKM and (DOPA)4-PEG5-YGFGG mixed with PBS solution (0.01 mg/ml) to modify the biosurface (60 min, at room temperature). Then, peptide-coated Ti-based materials were washed with Milli-Q water to eliminate the unbound peptides. Before implantation, the materials were disinfected with 75% ethanol and dried again for storage, under sterile conditions.

### 4.2. Characterization of Bioactive Peptides

HPLC analysis was performed to separate and quantify the bioactive peptide. The mass determination of ionized peptide was analyzed by ESI-MS. The molecular structure and dynamic chemical reactions were observed by nuclear magnetic resonance (NMR). QCM-D (Q-sense AB, Sweden) was used to quantify the mass of the molecules bound to the prefabricated coatings as previously described [30].

In addition, the two bioactive peptide coatings on Ti plates were characterized by AFM (Dimension ICON, Bruker, USA). Moreover, the surface chemical composition of the two bioactive peptide-modified Ti substrates was measured by XPS (EscaLabXi, Thermo Scientific, New York). The WCA measurement using a 4 *μ*l deionized water droplet. The surface hydrophilicity was measured with 4 *μ*l of deionized water by the sessile drop method on a contact angle system (Attension Theta Flex, Biolin Scientific, Sweden). For accurate statistics, we performed four measurements on different samples of each group.

### 4.3. Cell Culture, Isolation, and Characterization

In this work, osteoporosis BMSCs (op-BMSCs) were isolated from senile rats (male, 18 months old) that were previously confirmed to have osteoporosis by micro-CT imaging. The isolated op-BMSCs were maintained and expanded in *α*-MEM (VivaCell, Shanghai), which contains 10% fetal bovine serum (FBS, VivaCell, USA). Three to six passages were performed in these studies after identifying immunophenotypic markers by flow cytometry. Meanwhile, the Chinese Academy of Sciences provided normal BMSCs. These cells were cryopreserved in CELLSAVING (NCM biotech).

### 4.4. Biocompatibility Analysis

The biocompatibility analysis of different Ti-based surfaces was first assessed using a live/dead cell staining kit (Dojindo Laboratories, Japan). Staining was performed using calcein AM and PI at concentrations of 2 *μ*mol/l and 4.5 *μ*mol/l, respectively. As a result, the dead and live cells were stained red and green, respectively, under fluorescence microscopy (Zeiss). In addition, TRITC-labeled phalloidin (Yeasen, Shanghai, China) was used to show cytoskeletal actin with a fluorescence microscope. For SEM analysis, cells of sample surfaces were dehydrated by ethanol gradient dehydration for 5 min and then vacuum dried, sprayed with gold, and analyzed with an electron microscope. Image-Pro Plus 7.0 software was used to analyze the average cell spreading area.

Additionally, the cell adhesion forces on the peptide coating and smooth titanium were measured following an established method [42]. In brief, op-BMSCs were cultured on Ti surfaces in 24-well plates (4 × 10^4^ cells/well). The sample surface was gently rinsed with PBS after 6, 12, 18, and 24 h to remove the floating cells. The adhered cells on the surface were first counted before centrifugation. Additionally, the number of adhered cells after centrifugation was counted. The relative adhesion forces were calculated by the fraction of adhered cells after centrifugation at 600 rpm and 1000 rpm for 5 min.

### 4.5. In Vitro Cell Recruitment

Recruitment of op-BMSCs by peptide coating was investigated by transwell chambers. First, 6 groups of samples were placed at the bottom of a 24-well plate, and the cells were seeded on the upper chamber in *α*-MEM (5 × 10^3^ cells) for 12 h. Then, the upper chamber bottom membrane was fixed and further stained with 0.5% crystal violet (Beijing Leagene Biotechnology Co., Ltd. (Beijing, China)). Cells that did not penetrate the membrane were wiped off with a cotton swab before microscopic observation.

To evaluate competitive adhesion between BMSCs and fibroblasts (FBs, RFL-6), BMSCs and fibroblasts were cocultured. Briefly, FBs were prelabeled with Celltracker CM-DiI (red), while op-BMSCs were prelabeled with Celltracker CMFDA (green) in accordance with the manufacturer's instructions (Molecular Probes/Yeasen, China). After that, fluorescently labeled FBs and op-BMSCs were centrifuged for 5 minutes at 1200 rpm, followed by isolation with 0.25% trypsin solution (NCM Biotech, China).

The op-BMSCs and FBs were resuspended in *α*-MEM medium (with 10% FBS) to a concentration of 1 × 10^5^ cells/ml. Then, the cells were mixed in a volume of 1 : 1 and seeded onto the samples. After 12 h of culturing, the attached cells were obtained. A fluorescence microscope was used to observe attached cells.

### 4.6. In Vitro Osteogenic Differentiation

The osteogenic capacity of op-BMSCs was determined by alkaline phosphatase (ALP) staining and Alizarin Red (ARS) staining. Briefly, op-BMSCs (2 × 10^4^ cells/well) were cultured in osteogenic differentiation medium (HUXMX-90021, Cyagen) for 7, 14, and 21 days. For ALP staining, after rinsing with PBS, the cells were fixed in 4% paraformaldehyde for 15 min on ice. Furthermore, the prepared BCIP/NBT (Beyotime, China) working solution was used to stain the cells for 30 min. Meanwhile, ALP activity was detected using an ALP detection kit (NJJCBIO, Nanjing, China) at indicated time. First, trypsinize and centrifuge to collect cells on the surface of titanium plates in each group, add 80 *μ*l of radioimmunoprecipitation assay buffer to lyse the cells, and centrifuge at 14,000 r.p.m. for 10 minutes. Then, prepare a 96-well plate, according to the instructions, add 50 *μ*l of buffer and 50 *μ*l of matrix solution to all wells in turn, add 30 *μ*l of deionized water to blank wells, add 30 *μ*l of control solution to standard wells, and add 30 *μ*l of test sample to assay wells. Color development was stopped after 15 min incubation at 37°C, and absorbance was measured at 405 nm. In addition, the protein concentration of the samples was determined by the biscinconic acid (BCA) method, and finally, the ALP activity was calculated according to the measured absorbance value and protein concentration.

For ARS staining, the samples were first fixed in 95% alcohol for 20 min. Next, the cells were gently rinsed and incubated in ARS staining solution (Sigma, USA) for 30 min at pH 4.2. After taking pictures, calcium nodules were dissolved with 5% perchloric acid (Sigma-Aldrich, USA) for 30 min at room temperature. The absorbance was analyzed at 420 nm by a microplate reader.

### 4.7. qRT–PCR

The gene expression of cells was detected by SYBR Green mix (Vazyme Biotech Co., Ltd.). Briefly, total RNA was extracted by TRIzol reagent (Beyotime). The RNA density was quantified using a NanoDrop 2000 spectrophotometer (Thermo Fisher Scientific). Then, total RNA was reverse transcribed to obtain complementary DNA (cDNA). Next, cDNA was amplified using a BIO-RAD CFX96 touch q-PCR system. The mRNA level was calculated using a comparative 2-*ΔΔ*Cq method. All the primers are listed in Table S1.

### 4.8. Animals and Screw Implantation

The experimental procedures were licensed by the Animal Ethics Committee of Soochow University. A total of fifty 18-month-old male Sprague-Dawley (SD) rats were housed under climate-controlled conditions and fed freely. Among them, a total of 24 SD rats were randomly divided into four groups (*n* = 6): the control group, DOPA-E7 group, DOPA-Y5 group, and DOPA-(E7+Y5) group. All rats accepted Ti screw implant insertion in the femur after anesthetization through pentobarbital (Nembutal, 3.5 mg/100 g, i.p.). Briefly, bilateral knees were shaved and disinfected for the preparation of Ti-based screw implantation at the condyle of the distal femur. The drilling site was 0.5 cm proximal to the tangent of the distal femur. Ti-based screws coated with or without bioactive peptides were implanted in the bilateral femoral condyles by penetrating the double bone cortex. After that, the incision was sutured and closed in layers. In addition, all rats were intramuscularly administered 10 mg/kg calcein and alizarin red at 20 and 10 days before euthanasia. The duration of healing was 8 weeks.

### 4.9. Micro-CT Analysis

To evaluate the osseointegration capacity of the implant, the left femurs with implants (*n* = 6 per group) were scanned by micro-CT (SkyScan 1176, BRUKER, Belgium) at 9 mm resolution at the study endpoint. The specimens were scanned at a voltage of 60 kV and a current of 500 *μ*A with an 18 *μ*m voxel size. The rotation step was 0.7 degrees. A region of interest located surrounding the longitudinal axis of the screws between the outer diameter and inner diameter was chosen for the assessment. Three-dimensional reconstructed images were obtained for each specimen, and morphometric parameters were evaluated by the supporting software (CT Analyzer Version 1.10; SkyScan).

### 4.10. Histomorphometric Analysis

The collected femurs containing the screws were fixed in 4% paraformaldehyde. Parts of the femurs were embedded for undecalcified sectioning with a thickness of 50 *μ*m. Then, calcein/arizarin red double-labeling and Van Gieson staining (ACMEC, China) were performed to determine the dynamic bone formation and percentage of bone tissues contacting the implant. Parameters including the new bone area was assessed using the Bioquant Osteo 2017 system. Part of the femurs were decalcified, and Ti screws were carefully removed before embedding. Then, the specimens were sliced into 6 *μ*m sections for histologic staining. The general view of tissues was observed by H&E staining and Masson's staining, and pictures were visualized and captured through an AxioCam HRc microscope (Carl Zeiss).

The detection of BMSC migration-related markers was performed using immunofluorescence staining. In brief, tissue sections (implanted for one week) were incubated in primary antibodies including anti-STRO-1 (14-6688-82, eBioscience) and anti-CD146 (0806-5, HUABIO), for 12 h on ice after sealing. Then, the tissue sections were incubated with fluorescent secondary antibodies (Alexa Fluor®488, Abcam). Meanwhile, tissue sections were incubated in osteogenesis-related primary antibodies, including Col1A1 (A1352, ABclonal) and Runx2 (A2851, ABclonal)), and immunofluorescently visualized using fluorescent secondary antibodies (Alexa Fluor® 647, Abcam). Then, DAPI solution was added to counterstain the nucleus for 10 min. After that, the fluorescence of tissue sections was analyzed.

### 4.11. Statistical Analysis

All data are presented as the means ± SEM with at least three replicates for every experimental sample. Student's *t* test was used to analyze comparisons between two groups, and one-way analysis of variance (ANOVA) was used to analyze multiple comparisons. The data analysis was performed using SPSS 25.0 (USA). Statistical significance was defined as a *P* value less than 0.05.

## Data Availability

Data supporting the findings of this study can be obtained from the corresponding author upon request.

## Supplementary Materials

Supplementary Materials Figure S1: high-performance liquid chromatography spectrum of DOPA-E7 and DOPA-Y5 peptide. Figure S2: establishment and evaluation of aged osteoporotic rats. Figure S3: characterization of op-BMSCs. Figure S4: comparison of the proliferation, migration, and osteogenic differentiation of BMSCs in normal and osteoporotic conditions. Figure S5: biocompatibility of biomimetic peptide coatings. Figure S6: cell cytotoxicity and adhesion assay of different surfaces. Figure S7: the cell adhesion behavior on different surfaces. Figure S8: osteoblast-related gene expression of ALP, Runx2, Col1A1, and OCN on different peptide-treated surfaces. Figure S9: establishment of osteoimplant model in aged osteoporotic rats. Figure S10: RNA-sequence analysis. Figure S11: KEGG enrichment analyses revealing the top 25 pathways in op-BMSCs seeded onto the surface of the peptide coating. Figure S12: screening out hub genes of the top 25 KEGG pathways. Table S1: summary of the forward and reverse gene primers used in real-time PCR.
