# Supplementary material for: Engineering Stem Cell Recruitment and Osteoinduction via Bioadhesive Molecular Mimics to Improve Osteoporotic Bone-Implant Integration
Source: Research (Wash D C). 2022 Sep 6;2022:9823784. doi: 10.34133/2022/9823784 (PMC9484833; doi:10.34133/2022/9823784)
Supplement: Supplementary Materials — Figure S1: high-performance liquid chromatography spectrum of DOPA-E7 and DOPA-Y5 peptide. Figure S2: establishment and evaluation of aged osteoporotic rats. Figure S3: characterization of op-BMSCs. Figure S4: comparison of the proliferation, migration, and osteogenic differentiation of BMSCs in normal and osteoporotic conditions. Figure S5: biocompatibility of biomimetic peptide coatings. Figure S6: cell cytotoxicity and adhesion assay of different surfaces. Figure S7: the cell adhesion behavior on different surfaces. Figure S8: osteoblast-related gene expression of ALP, Runx2, Col1A1, and OCN on different peptide-treated surfaces. Figure S9: establishment of osteoimplant model in aged osteoporotic rats. Figure S10: RNA-sequence analysis. Figure S11: KEGG enrichment analyses revealing the top 25 pathways in op-BMSCs seeded onto the surface of the peptide coating. Figure S12: screening out hub genes of the top 25 KEGG pathways. Table S1: summary of the forward and reverse gene primers used in real-time PCR. [file 9823784.f1.docx]

**Engineering Stem Cell Recruitment and Osteoinduction via Bioadhesive Molecular Mimics to Improve Osteoporotic Bone-Implant Integration**

Jiaxiang Bai^1,2^, Gaoran Ge^1^, Qing Wang^1^, Wenming Li^1^, Kai Zheng^1^, Yaozeng Xu^1^, Huilin Yang^1^, Guoqing Pan^2,^* and Dechun Geng^1,^*

* **Correspondence should be addressed to** Guoqing Pan; panguoqing@ujs.edu.cn and Dechun Geng; [szgengdc@suda.edu.cn](mailto:szgengdc@suda.edu.cn)

^1^ Department of Orthopaedics, The First Affiliated Hospital of Soochow University, 188 Shizi Street, Suzhou, Jiangsu 215006, P. R. China.

^2^ Institute for Advanced Materials, School of Materials Science and Engineering, Jiangsu University, 301 Xuefu Road, Zhenjiang, Jiangsu 212013, P. R. China.

**This PDF file includes:**

Supplementary Text

Figures. S1 to S11

Tables S1

Supplementary Text

Figure S1. High-performance liquid chromatography spectrum of DOPA-E7 and DOPA-Y5 peptide.

Figure S2. Establishment and evaluation of aged osteoporotic rats.

Figure S3. Characterization of op-BMSCs.

Figure S4. Comparison of the proliferation, migration and osteogenic differentiation of BMSCs in normal and osteoporotic conditions.

Figure S5. Biocompatibility of biomimetic peptide coatings.

Figure S6. Cell cytotoxicity and adhesion assay of different surfaces.

Figure S7. The cell adhesion behavior on different surfaces.

Figure S8. Osteoblast-related gene expression of ALP, Runx2, Col1A1, and OCN on diﬀerent peptide-treated surfaces.

Figure S9. Establishment of osteoimplant model in aged osteoporotic rats.

Figure S10. RNA-sequence analysis.

Figure S11. KEGG enrichment analyses revealing the top 25 pathways in op-BMSCs seeded onto the surface of the peptide coating.

Figure S12. Screening out hub genes of the top 25 KEGG pathways.

Table S1. Summary of the forward and reverse gene primers used in real-time PCR.

**Figure S1:** **High-performance liquid chromatography spectrum** of (**a**) DOPA-E7 and (**b**) DOPA-Y5 peptide.

**Figure S2:** **Establishment and evaluation of aged osteoporotic rats.** (**a**) 3D representative micro-CT images of femoral and trabecular bone. (**b**) Quantitative parameters of morphological bone alterations, including BMD, BV/TV, Tb. Th, and Tb. Sp in the ROI. (n=3; **P < 0.005 and ***P < 0.001 compared with the normal control group).

**Figure S3:** **Characterization of op-BMSCs.** (**a**) FCA was used to analyze cell marker expression on the surface of op-BMSCs. (**b**) Quantitative analysis of the expression of cell markers on the surface of op-BMSCs.

**Figure S4:** **Comparison of the proliferation, migration and osteogenic differentiation of BMSCs in normal and osteoporotic conditions.** (**a**) CCK-8 assay at the indicated time points. (**b**) Images of scratch experiment at the indicated time points. (**c**) ALP staining, ARS staining and corresponding quantification. (n=3; *P < 0.05, **P < 0.005 and ***P < 0.001 compared with the normal BMSCs group).

**Figure S5:** **Biocompatibility of biomimetic peptide coatings.** (**a**) Live/dead staining of op-BMSCs on the bare and modified Ti-based surfaces (4:0, 3:1, 2:2, 1:3, and 0:4) for 24 and 72 h. (**b**) Quantification of dead cells per field. (n=5; ***P < 0.001 compared with the bare surface group).

**Figure. S6.** **Cell cytotoxicity and adhesion assay of different surfaces.** (**a**) Cell cytotoxicity of op-BMSCs on different surfaces for 24, 48 and 72 hours. (**b**) Results of the cell adhesion assay for 2 and 4 hours. (n=5; **P < 0.005 and ***P < 0.001 compared with the bare surface group).

**Figure S7:** **The cell adhesion behavior on different surfaces.** (**a**) Cytoskeleton staining (TRITC-phalloidin/DAPI) of op-BMSCs on different surfaces. (**b**) Scanning electron images of op-BMSCs on different surfaces. (**c**) Quantification of the average cell area. (n=5; ***P < 0.001 compared with the bare surface group).

**Figure S8:** **Osteoblast-related gene expression** of (**a**) ALP, (**b**) Runx2, (**c**) Col1A1, and (**d**) OCN on diﬀerent peptide-treated surfaces. (n=5; **P < 0.005 and ***P < 0.001 compared with the bare surface group).

**Figure S9.** **Establishment of osteoimplant model in aged osteoporotic rats.**

**Figure S10.** **RNA-sequence analysis.** (a) RNA-sequence analysis of op-BMSCs on bare Ti and peptide-coated Ti surfaces (0:0 and 2:2). (**b**) GO classification showing the functions of differentially expressed genes. (**c**) GSEA of genes associated with chemokine-mediated signaling pathways.

**Figure S11**. **KEGG enrichment analyses revealing the top 25 pathways in op-BMSCs seeded onto the surface of the peptide coating.**

**
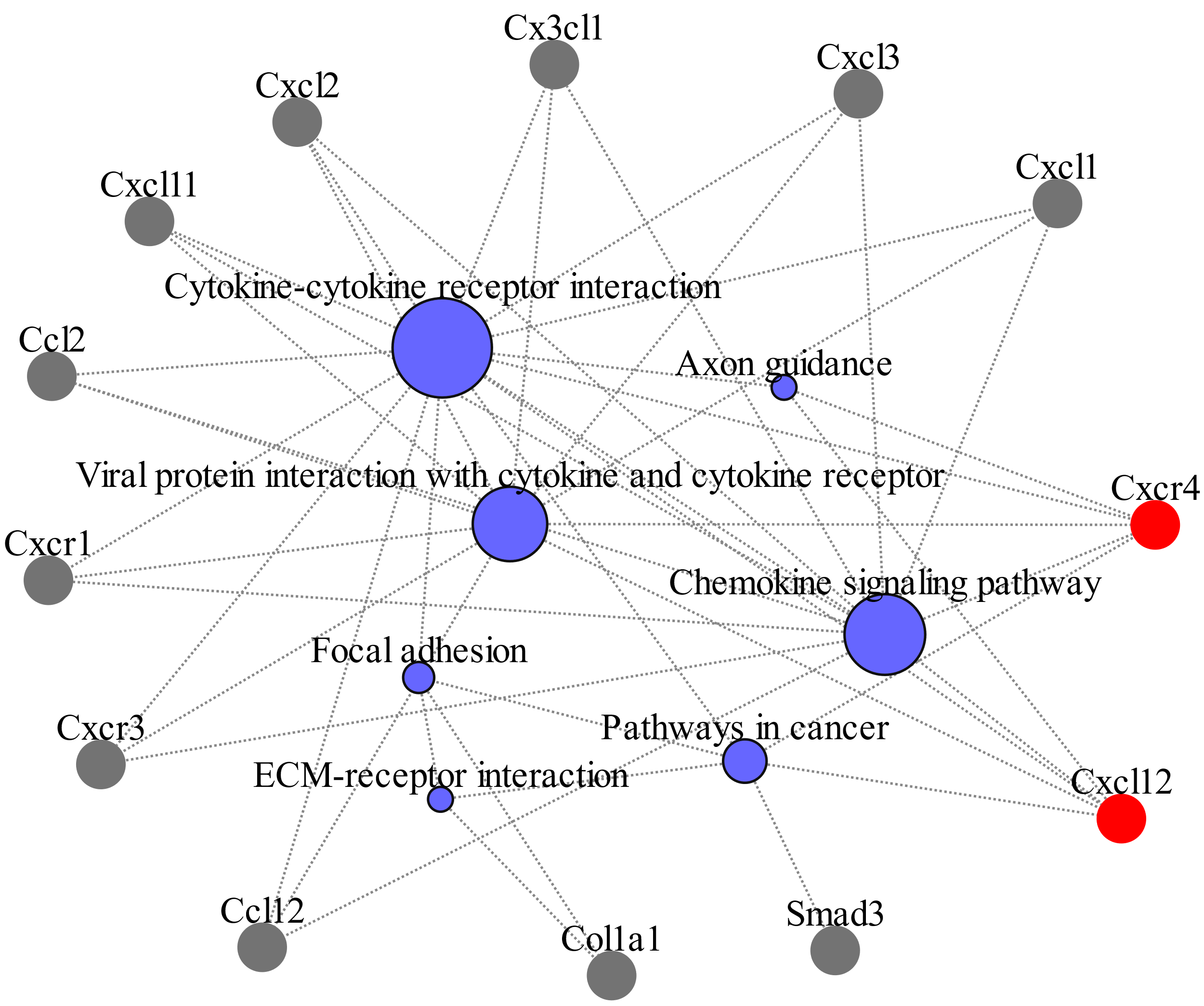
**

**Figure S12**. **Screening out hub genes of the top 25 KEGG pathways.**

**Table S1.** **Summary of the forward and reverse gene primers used in real-time PCR.**

| **Gene** | **Forward primers** | **Reverse primers** |
| --- | --- | --- |
| *Alp* | ATGCTCAGGACAGGATCAAA | CGGGACATAAGCGAGTTTCT |
| *Runx2* | ATCATTCAGTGACACCACCA | ATCATTCAGTGACACCACCA |
| *Col1A1* | AGCTCGATACACAATGGCCT | CCTATGACTTCTGCGTCTGG |
| *Ocn* | CAGACAAGTCCCACACAGCA | CCAGCAGAGTGAGCAGAGAG |
| *Cxcl12* | GAGCCAACGTCAAACATCTGAA | TCCAGGTACTCTTGGATCCACTTTA |
| *Cxcl6* | CTTAGCTCCAAGAATTAACC | GGTCAAGACAAACATTATCC |
| *Cxcr4* | AGTGACCCTCTGAGGCGTTTG | GAAGCAGGGTTCCTTGTTGGAGT |
| *β-actin* | CCTCTATGACAACACAGT | AGCCACCAATCCACACAG |
